# Susceptibility to Disease

**Published:** 1899-01

**Authors:** F. S. Davis

**Affiliations:** Quincy, Mass.


### SUSCEPTIBILITY TO DISEASE.

F. S. DAVIS, M. D., QUINCY, MASS.

Not long ago I was talking with two brothers who told me they had been sailors in previous years, and had been in many climes and exposed to many diseases. They had both cared for small-pox and yellow fever patients, and handled the bodies of those who had died from these diseases.

They had never been sick. These examples of immunity brought forcibly to my mind the subject of susceptibility to disease, which I have taken for discussion.

The questions, why and how do people get sick, come up for answer.

In the case of these brothers there is no question that their systems were exposed to the contagion of these diseases. Why were they not made sick? There must be a logical reason why a disease did not develop in their system.

In making a study of man in health and disease the better to understand why it is that sickness comes and how it comes, the true follower of Hahnemann must become acquainted with something more than the anatomy, physiology, and the pathology of the body of man. It is essentially necessary that he also become master of the laws controlling the force which is the life of the body—the vital force, as it is called. Observation teaches that all matter has a force stored up in it, and that force constitutes its life. It would be impossible for any matter to exist without its individual force.

All substances known to man have the power to react when properly acted upon. The most inert substances may thus become active, and when the conditions are right will develop the characteristics which make them what they are. Some substances easily become active, others remain inert until they are properly called upon, when the bands of their captivity are broken and they respond to the call, and in a wonderful way develop the force that is their life, imparting it to matter which they come in contact with. But before this action of giving and receiving can take place there must be a condition favorable for the changes.

The force to be imparted must be in the right stage or degree of activity, corresponding to a certain activity in the receiving body.

We find by observing the action of the human system that it is, under certain conditions, in a state when it is possible for it to receive that which proves an injury to it, and the result is disease.

We learn from *The Organon* what it is that is disturbed, and how it affects the body of man. Permit me to read a few sections touching upon this point.

"SEC. 9. In the healthy condition of man, the immaterial vital principle, which animates the material body, exercises an absolute sway, and maintains all its parts in the most admirable order and harmony, both of sensation and action, so that our indwelling rational spirit may fully employ these living, healthy organs for the superior purposes of our existence.

"SEC. 10. The material organism, deprived of its vital principle, is incapable of sensation, action, or self-preservation; it is the immaterial vital principle only, animating the former in its healthy and morbid condition, that imparts to it all sensation, and enables it to perform its functions.

"SEC. 11. When a person falls ill it is only the spiritual, self-acting (automatic), vital force, everywhere present in his organism, that is primarily deranged by the dynamic influence upon it of a morbific agent inimical to life; it is only the vital force, deranged to such an abnormal state, that can furnish the organism with its disagreeable sensations, and incline it to the irregular processes which we call disease; for, as a power invisible in itself, and only cognizable by its effects on the organism, its morbid derangement only makes itself known by the manifestation of disease in the sensations and functions of those parts of the organism exposed to the senses of the observer and physician—that is, by *morbid symptoms*.

"SEC. 12. It is the morbidly-affected *vital force* alone that produces disease. . . ."

Now, the question next to be answered is, Why should the vital force of one man become disturbed, and not every man, when exposed to disease-producing causes? Here we come to a very important point, one which has been overlooked, or at least unduly considered. It is *the susceptibility*, or, as it is sometimes called, predisposition to disease.

There are so many points which come to view as we look over the history of man that have a bearing upon this question, that it is a difficult matter to select a few for consideration, as many have great weight.

Dr. Kent says that man originally never was sick. Then it seems to me he could not have been at that time susceptible to influences that now make him sick; or if man has not changed in this respect, new enemies have come, and are very busy doing effective work against man. We do not find healthy people to-day. Seldom do we find a child in perfect health at birth. This is a startling observation when we come to think that there was a time when man was in perfect health.

Dr. Fincke, in his work on *High Potencies,* says: "It must be admitted that we know nothing positively of the nature of health, disease, and remedy, and that their proportions and existence are only inferred from the effects they produce in the organism. Equally so, we know nothing positively of the nature of things and forces generally."

He quotes Draper as follows:

"There is no essential difference between the processes of organic and inorganic life, and the line of demarcation which natural history has, so far, vainly attempted to define with correctness between organic and inorganic world is merely arbitrary: Either of them is reducible to motion, and governed by the same laws." Fincke says: "Hahnemann and his greatest disciples always stoutly maintained the *hylozoic* opinion that everything in nature lives, and this is confirmed by the nature, properties, and efficaciousness of our high potencies."

Thus it is as Joslin says (*Principles of Homœopathy,* 1850): "It is the destiny of Homœopathy not only to effect a glorious revolution in the art of healing, but to lead to new views of the constitution of matter."

One of the most important views opened up by Homœopathy is that all we can see in the world are results. Homœopathy applies this even to disease, holding it to be, so far as we ever see it, a result. No one ever sees causes. No microscope will ever find them. We only see the effects of force; never the force itself.

Homœopathy demonstrates the fact that everything that is capable of affecting the processes of man's economy, to produce disease or to cure disease, works in accordance with the Law of Similars, and dynamically, and confirms the doctrine of Hippocrates that "each disease has its own peculiar process, is produced by the similia, and by the administered similia from being sick they get well."

From this law of action we must get our information of how and why people get sick. We have only to recognize the mutual action in the case, which is clear, as Dr. Fincke observes. Mutual action consists of action and reaction, and they are always contrary and equal under the third Newtonian law—contrariety and equality of mutual action.

We must, I believe, consider the sick-making and the sick-curing power of a drug the same action, only bearing in mind that the difference between the two is of conditions, not of things or principles, and we must hold to the same in our views of the action of miasms which produce true diseases. They must act as drugs do. The observations of Dr. Fincke help us here. "*Mutuality of action* is the qualitative character of all medical action. For the purpose of effecting the cure, as the organism must be susceptible of the remedy, so the remedy must be susceptible by the organism and its concerning organs. The susceptibility of the organism is varying and different in each individual case.

"For this reason it is necessary to individualize the susceptibility as well as the dose, and the remedy. The *susceptibility* serves as the diagnostic principle of Homœopathy. Susceptibility depends upon the assimilability of matter in general. As the organism is assimilative to the remedy, so the remedy is assimilable by the organism, and *vice versa*.

"Pathologically, as well as physiologically, life is conditioned by assimilation, and it depends upon assimilation in disease as well as in health, and this much was taught by Hippocrates. Health and disease are not contradictory things, but, as has been observed by Hahnemann, Reil, and Comte, contrasted forms and modes of existence, and contrary states of the same organism, and both are governed by the same laws."

Now if, as these observations prove, there is a susceptibility to account for the action of the remedy, and if disease is produced by and cured by similars, then it follows that disease is produced by something to which the system is susceptible. It follows naturally that if there was no susceptibility there could be no disease. Hence, in a sense, it is true that man would not be sick unless he were already ailing, or, as we may express it, if he had no sickness within him he would not get sick externally. Therefore, to really cure a man he must get rid of the sickness within him—that is, the susceptibility.

Now let us consider this susceptibility which is the starting point of disease. Where did it come from? Where did it get so as to produce its state in man?

We have learned from Hahnemann and others that disease is a result of a dynamic disturbance of that spirit-like life force that is the life controller in man. Therefore, it must be that this variation from the normal state of health, which must exist before man can be truly sick, consists of a disturbance in this automatic regulator. This is termed by Hahnemann the fundamental cause of disease, due to a chronic miasm. He names three: psora, syphilis, and sycosis, psora being the greater.

Dr. Kent says psora is "the cause of all contagion." He says "if man had not had psora he would not have had the other two chronic miasms, but psora, the oldest, became the basis of the others," and Hahnemann teaches that acute diseases arise because of the existence in the system of the chronic.

I believe it is Dr. Kent who has said that the susceptibility to disease was all there was to be really cured to make a patient well.

We must conclude from all these observations that disease comes to man because of a defect in the working of the vital force, and that is entirely a dynamic process, proceeding from the interior of man, and then manifesting itself visibly externally.

DISCUSSION.—DR. DAVIS'S PAPER, "SUSCEPTIBILITY TO DISEASE."

Dr. Kimball—We often see curious phases of this immunity to or from disease. I know of the case of a physician who has been through three epidemics of small-pox without taking it, and who has been vaccinated nineteen times without its taking effect. It seems as though he had no susceptibility to the acute miasm known as small-pox. Yet he is not a well man, for two or three years ago he nearly died of pneumonia; so there is in him the presence of a psoric miasm. Possibly, if he were subjected to some prolonged mental strain he might then be susceptible to small-pox. Of course, when we speak of the original cause or source of disease, man being in his first state a healthy being, it raises a great question, but it is undoubtedly true that the mental condition of man gave rise to the first diseased condition.

Dr. Pease—Dr. Davis quoted Hahnemann, and probably correctly, as speaking of the self-acting, automatic vital force. How can that be possible? This you must recognize: that there must be an energy back of it. Can we get away from that condition? Back of every identity there must be an essence. Take another statement of Hahnemann's in regard to the question. He makes certain observations on treatment of the cause, or treatment founded on the internal essence of the disease. He divides diseases into two classes—that is, diseases having a simple, material cause, and diseases having an immaterial, dynamic cause. Now this so-called material cause of disease could not be a cause or bring about the effects if there was not an essence back of it. We give a drug in repeated doses or a single dose, and if the essence of that drug, or its vital principle, is inimical to the condition which we call health in the individual receiving the drug, there will be a disturbance, and the symptoms resulting are the visible signs of the inimical power which produces the disease and the susceptibility of the patient. In regard to the law of action and reaction the writer spoke of: In the first place, what is a law? We see it illustrated in the action of Belladonna, or the force we call Belladonna. It operates in accordance with the law which governs, or presides over, or was placed in action at the time of the first creative manifestation of the Belladonna essence, and for all time the operation of Belladonna must be under the control of that law. Another substance that is contrary to Belladonna may act as a dynamic antidote. Is that antidotal action, which is dynamical, under the law of similars or the law of opposites? I think that as homœopathic physicians we should study these questions.

Dr. Kimball spoke of the condition of the mind as being the cause of the first man having his first sickness. That harmonizes with my paper of last year, because just so long as there is sin in the world will there be work for doctors to do, and room for the law of Homœopathy to be put into active use. The cure of disease by the application of drug power, must be in accordance with the laws of affinity, or the laws of repulsion, and it is our place to study into and possibly learn these laws. The only way to do that is to discriminate between the essence of a drug, and its material manifestation in visible form.

Dr. Kennedy—Health and disease are relative terms. With that in mind I think I will differ somewhat from a statement made in the paper—that seldom is a child born into the world healthy. I was interested in the paper, and interested in the idea that it is the susceptibility which must be removed in order that the patient may be restored to health. Our potentized remedies, given homœopathically, so act that the patients—and we treat patients, not diseases—are restored to comparative health. Oftentimes, as we have all verified, a person, after being treated homœopathically, remains well for years. Dr. Kimball suggested the idea that a man may be susceptible in one direction and not in another. For instance, a man may be in good health, but so susceptible to a certain substance that, upon coming in contact with it, even in a very slight degree, he will become seriously affected in health.

Dr. Pease said: "How can the vital force act automatically without a power behind it?" No life can act without the one power behind it. I believe that with all our philosophy, and with all our attempting to analyze and define life, we must fall back upon that one point, that back of all of it is the power that keeps all things in the universe alive—the Almighty.

Dr. Patch—I do not understand from *The Organon* that Hahnemann, in speaking of the self-acting, automatic vital force, implied that the Almighty power was not behind the vital force. I look upon the vital force as that wonderful balance acting between the higher, spiritual nature, and the lower, material part of the system, and of course if it is such it may be automatic or self-acting, and still be prompted by the spiritual nature of man behind.

Dr. Adams—Could the vital force be anything else than automatic? How could it be anything else?

Dr. Close—If it is spiritual force then it *is* automatic, having the source of action in itself.

Dr. Patch—Are we not confusing the spiritual and dynamical, perhaps unthinkingly? The spirit in man prompting the vital force is not necessarily Divinity, but the agent of Divinity.

Dr. Davis—You are considering the mental state. I had a mind to go into that but it would have made my paper too long. We recognize the mental state as being the highest, as Hahnemann did in all of his teachings. Mental symptoms are the most important as representing the highest plane in man, and being most indicative of man's nature. Dr. Kent covers the same ground somewhere in his writings: that man predisposed himself first to disease by wrong living. He thought wrong, and his thoughts gave rise to wrong actions, and these wrong actions went on to undermine his will to do right. He got into wrong ways from the start.

About the law of action and reaction spoken of by one of our members, who questioned as to whether the reaction was opposite. It must be opposite, for action and reaction are equal and opposite. Dr. Kennedy thinks that there are children born healthy. If disease depends upon susceptibility, and the susceptibility exists, then they are not well. There is the real weakness of the disease to begin with. In that case there are few children born healthy. This susceptibility is inherited from their ancestors. They had nothing to do with it; they were born with the susceptibility. In that sense they are not well. Kent says that the only way to make people well is to cure the susceptibility. We know that if the mothers, and we may say fathers, also, are treated before conception, the child will be more healthy, but if they are not born that way I think few are well. It would be a great question to consider, as to whether diseases are governed by vibration or motion of the small particles which make up the substance of man. I think it must be so. I have nothing more to say in closing the discussion of my paper.

---

Rumex Crispus.—If there is one remedy which is of benefit in most coughs it is Rumex crispus. The yellow dock has many symptoms reminding one of Pulsatilla, but its aggravation on inspiration is characteristic of Rumex alone.—*Medical Visitor*, Vol. XIV, No. 8.

